# Estimating the health consequences of flight attendant work: comparing flight attendant health to the general population in a cross-sectional study

**DOI:** 10.1186/s12889-018-5221-3

**Published:** 2018-03-23

**Authors:** Eileen McNeely, Irina Mordukhovich, Samuel Tideman, Sara Gale, Brent Coull

**Affiliations:** 1 Department of Environmental Health, Harvard T.H. Chan School of Public Health, Boston, MA USA; 2 Department of Biostatistics, Harvard T.H. Chan School of Public Health, Boston, MA USA

**Keywords:** Flight attendants, Occupational epidemiology, Cancer, Depression, Fatigue

## Abstract

**Background:**

Flight attendants are an understudied occupational group, despite undergoing a wide and unique range of adverse job-related exposures. In our study, we aimed to characterize the health profile of cabin crew relative to the U.S. general population.

**Methods:**

In 2014–2015, we surveyed participants of the Harvard Flight Attendant Health Study. We compared the prevalence of their health conditions to a contemporaneous cohort in the National Health and Nutrition Examination Survey (NHANES 2013–2014) using age-weighted standardized prevalence ratios (SPRs). We also analyzed associations between job tenure and selected health outcomes, using logistic regression and adjusting for potential confounders.

**Results:**

Compared to the NHANES population (*n* = 2729), flight attendants (*n* = 5366) had a higher prevalence of female reproductive cancers (SPR = 1.66, 95% CI: 1.18–2.33), cancers at all sites (SPR = 2.15, 95% CI: 1.73–2.67 among females), as well as sleep disorders, fatigue, and depression, with SPRs ranging between 1.98 and 5.57 depending on gender and the specific condition examined. In contrast, we observed a decreased prevalence of cardiac and respiratory outcomes among flight crew relative to NHANES. Health conditions that increased with longer job tenure were sleep disorders, anxiety/depression, alcohol abuse, any cancer, peripheral artery disease, sinusitis, foot surgery, infertility, and several perinatal outcomes.

**Conclusions:**

We observed higher rates of specific adverse health outcomes in U.S. flight attendants compared to the general population, as well as associations between longer tenure and health conditions, which should be interpreted in light of recall bias and a cross-sectional design. Future longitudinal studies should evaluate specific exposure-disease associations among flight crew.

**Electronic supplementary material:**

The online version of this article (10.1186/s12889-018-5221-3) contains supplementary material, which is available to authorized users.

## Background

Flight attendants are an understudied occupational cohort, despite undergoing a wide and unique range of adverse job-related exposures. These workers are consistently exposed to cosmic ionizing radiation, circadian rhythm disruption due to night shift work and frequently crossing time zones, poor cabin air quality, elevated ozone levels, hypoxia, pesticides from cabin disinsection, high levels of occupational noise, heavy physical job demands, and verbal and sexual harassment [1–4]. Until 1998, they were also exposed in-flight secondhand tobacco smoke, especially before partial smoking bans were implemented in the year 1988 [5]. The long-term effects of this historical secondhand smoke exposure have not been well characterized.

Flight crew have historically been excluded from the Occupational Safety and Health Administration protections granted to most U.S. workers. Some limited protections were implemented in 2014 [6]. However, flight attendants’ exposure to ionizing radiation is not monitored, despite the National Council on Radiation Protection (NCRP) reporting that flight crew have the largest average annual effective dose of all U.S. radiation workers [7, 8].

The literature regarding flight crew health is relatively sparse and of varying quality [2]. Results have been mixed, but overall point towards associations between in-flight exposures or job tenure and reduced respiratory health [9], increased rates of breast and skin cancers [10], adverse reproductive and perinatal outcomes [11], musculoskeletal injuries [2], health effects from contaminated cabin air [12], and higher rates of mental health conditions [1, 13].

To address gaps in the existing literature (namely that (1) few U.S. studies have compared flight attendant health to that of the general population, especially for a comprehensive range of health outcomes, and (2) many previous studies of flight attendant health were of small sample size or prone to selection bias due to recruitment to investigate specific health issues), we launched the Harvard Flight Attendant Health Study (FAHS) in 2007 [14]. In the first wave of our study, we aimed to profile prevalent health conditions among cabin crew, compare the health of flight attendants to that of the general population, and evaluate associations between job tenure as a proxy for occupational exposures and prevalent health outcomes among cabin crew. This study reported elevated rates of reproductive cancers, respiratory and cardiovascular outcomes, and sleep and mental health conditions in flight attendants, some of which were related to job tenure [14]. We have since completed the second wave of the FAHS in 2014–2015, with new and returning participants. We aim to replicate our previous analysis with the goal of characterizing a possibly changing flight crew health profile given an older and more diverse work force, increased physical job demands, a longer time elapsed since the institution of in-flight smoking bans, and an updated fleet. We hypothesized that we would continue to observe associations between work as a flight attendant and a range of respiratory, cardiac, reproductive, perinatal, mental health and cancer outcomes.

## Methods

### Study population

Our participants were enrolled in the second wave of the FAHS, an ongoing study of flight crew health which was established in 2007 and originally enrolled 4011 flight attendants [14]. For the 2014–2015 wave of the FAHS reported in this manuscript, we recruited both new and returning flight attendants to participate through several channels, including a hard copy survey mailed to the 2007 participants and distributed at airport terminals between December 2014 and June 2015, and an online survey launched in December 2014. We supplemented our survey reach with in-person recruitment at five large airport hubs in the U.S. Our recruitment campaign also included announcements about the study from local unions and through social media. Survey participants could enter a lottery to win an iPad or Apple watch over an 18-month period.

Any current or former U.S. flight attendant was eligible to participate in the FAHS. We collected 1642 surveys from returning participants, which represents a 40% response rate from the original cohort with still-valid addresses. In total, the 2014–2015 FAHS cohort enrolled 5366 U.S. flight attendants with information on age and gender. These variables were among the last questions to be asked in the online questionnaire and are thus indicators of survey completeness. Our study was approved the Harvard T.H. Chan School of Public Health institutional review board, and all participants provided their written informed consent.

### Survey

Our survey instrument included validated questions about self-reported health outcomes and symptomology, work experiences, and personal characteristics [14], taken from established surveys such as the Job Content Questionnaire and the National Health and Nutrition Examination Survey (NHANES) [15, 16]. Participants were also asked to provide aviation employment history, including airlines, primary hubs, and dates of employment and leave.

### Comparison to NHANES

We compared the prevalence of health conditions and symptoms reported in the 2014–2015 wave of the FAHS to equivalent information collected from a nationally representative sample from the NHANES during the years 2013–2014 [16]. The NHANES is administered by the Centers for Disease Control and Prevention, and collects demographic, health, dietary, and biomarker data from approximately 5000 U.S. residents each year. Most of the health conditions we compared had binary answer choices for prevalence (ever diagnosed: yes/no). For fatigue and depression, prevalence was based on symptoms reported in the past two weeks in both the FAHS and NHANES surveys: we considered symptoms occurring “nearly every day” as a “yes” for both conditions. We weighted the NHANES data by their two-year sample weights, primary sampling units, and strata based on analytic guidelines [17], and restricted respondents to adults who had a family income to poverty ratio of 1 or greater, at least a high school education, and were currently employed (*N* = 2729) in order to better match our study populations.

### Statistical analyses

We calculated descriptive statistics for participant characteristics and health outcomes, and we compared participants taking hard copy and online questionnaires on key characteristics using Student’s *t* test and chi-square analysis. We then compared the prevalence of health behaviors, conditions and symptoms in the NHANES and FAHS using the Standardized Prevalence Ratio (SPR), an indirect method of standardization which compares observed and expected prevalence given rates in the reference study population, which in our case was NHANES [18]. The SPR was weighted by age category (18–39, 40–59, and 60+ years) and analyzed separately by gender.

To further increase the comparability of the study populations, we conducted a sensitivity analysis restricted to non-Hispanic white participants, who comprised 75% of our cohort and 43% of the NHANES population. We also conducted a secondary analysis evaluating the age-adjusted comparative prevalence of health conditions for flight crew exposed to high levels of historical occupational secondhand smoke, and we conducted sensitivity analyses which calculated SPRs for chronic bronchitis, coronary heart disease, reproductive cancer, and sleep disorders among participants taking hard copy surveys and among returning 2007 participants.

We also analyzed gender-stratified associations between net job tenure (total time working as a flight attendant minus any leave) and diagnosed health conditions using logistic regression and adjusting for potential confounders: age (continuous), current and past smoking status (yes/no), overweight status based on body mass index (25+ vs. < 25 kg/m^2^), and educational attainment (high school, some college/trade certificate, and college degree or higher). Tenure was meant to serve as a proxy for the duration of occupational exposures [19]. We examined perinatal and reproductive outcomes in relation to tenure prior to age 45. Analyses were completed using STATA statistical software, version 14 (StataCorp, College Station, TX).

## Results

FAHS participants (*n* = 5366) presented with a mean age of 51.5 years (Table 1). The average net job tenure was 20.4 years and 81.4% were female. Only 8.1% of the participants reported being current smokers; 32.7% were former smokers. Over 90% of participants completed at least some college or post-high school vocational training. Participants taking hard copy surveys differed from online survey takers in terms of age, tenure, sex, smoking status, and ethnicity, and were similar in terms of overweight, race, education, and past smoking history (data not shown).

**Table 1:** Characteristics by gender, Harvard Flight Attendant Health Study, 2014–2015

Characteristic	Female (*n* = 4368)		Male (*n* = 998)	
	Mean (SD)	No. (%)	Mean (SD)	No. (%)
Age (years)				
18–39		641 (14.7%)		215 (21.5%)
40–59		2349 (53.78%)		601 (60.2%)
≥ 60		1378 (31.6%)		182 (18.2%)
Tenure as flight attendant (years)	21.1 (11.8)		17.4 (10.1)	
Educational attainment				
< High school diploma/GED		1 (0.02%)		2 (0.2%)
High school diploma/GED		290 (6.9%)		56 (5.8%)
Some college/trade certificate		2088 (49.9%)		442 (46.0%)
College degree or higher		1808 (43.2%)		462 (48.0%)
Current smoker				
No		3899 (93.0%)		846 (87.7%)
Yes		295 (7.0%)		119 (12.3%)
Past smoker				
No		2872 (68.6%)		628 (65.2%)
Yes		1317 (31.4%)		335 (34.8%)
Overweight (BMI > 25 kg/m^2^)				
No		2530 (66.0%)		374 (41.1%)
Yes		1306 (34.1%)		536 (58.9%)

*BMI* Body mass index, *SD* Standard deviation

Acute and chronic health conditions reported by at least 15% of the FAHS population are presented in Table 2. These conditions fall primarily into the following categories: respiratory, musculoskeletal, cardiovascular, and mental health. Over 15% of study participants had also been diagnosed with any cancer. Except for high cholesterol and hypertension, the prevalence of the profiled conditions was consistently slightly higher among the female participants.

**Table 2:** Prevalence of health conditions and symptoms reported by at least 15% of participants

	All participants	Female	Male
	No. cases (%)		
Frequent symptoms: lasting 5–7 days over the past week			
Dry eyes	1036 (20.0%)	890 (21.2%)	146 (15.1%)
Sinus congestion	1279 (24.7%)	1033 (24.5%)	246 (25.3%)
Sinus pain	867 (16.8%)	710 (17.0%)	157 (16.3%)
Runny nose	1064 (20.6%)	901 (21.4%)	163 (16.9%)
Bloating	1291 (25.0%)	1117 (26.6%)	174 (18.0%)
Fatigue	1392 (25.9%)	1156 (26.5%)	236 (23.7%)
Anxiety	845 (16.4%)	696 (16.6%)	149 (15.4%)
Sleep disturbances	1838 (35.4%)	1541 (36.6%)	297 (30.6%)
Back pain	1243 (24.0%)	1051 (24.9%)	192 (19.8%)
Foot pain	1250 (24.2%)	1078 (25.6%)	172 (17.8%)
Shoulder/elbo*w*/wrist/hand pain	1531 (29.7%)	1323 (31.5%)	208 (21.5%)
Joint pain	1101 (21.3%)	949 (22.6%)	152 (15.7%)
Notable conditions: needing medical attention over the past year			
Dry eyes	1075 (21.4%)	909 (22.3%)	166 (17.7%)
Sinus congestion	2173 (42.7%)	1813 (43.9%)	360 (37.8%)
Sinus pain	1943 (38.3%)	1620 (39.2%)	323 (33.9%)
Ear pain	1339 (26.6%)	1145 (28.0%)	194 (20.6%)
Ear infection	755 (15.1%)	649 (16.0%)	106 (11.3%)
Runny nose	1132 (22.5%)	947 (23.1%)	185 (19.6%)
Sore throat	975 (19.4%)	814 (19.9%)	161 (17.0%)
Cough	1408 (27.8%)	1167 (28.4%)	241 (25.4%)
Hoarseness	790 (15.8%)	674 (16.6%)	116 (12.3%)
Bloating	862 (17.3%)	725 (17.9%)	137 (14.6%)
Fatigue	1076 (21.4%)	899 (22.0%)	177 (18.8%)
Anxiety	1070 (21.3%)	896 (22.0%)	174 (18.5%)
Depression	817 (16.3%)	667 (16.4%)	150 (15.9%)
Sleep disturbance	1364 (27.1%)	1133 (27.7%)	231 (24.4%)
Back pain	1376 (27.3%)	1145 (28.0%)	231 (24.3%)
Foot pain	1034 (20.6%)	878 (21.6%)	156 (16.5%)
Shoulder/elbow/wrist/hand pain	1463 (29.0%)	1254 (30.6%)	209 (22.1%)
Joint pain	849 (17.0%)	711 (17.5%)	138 (14.6%)
Pneumonia	1.380 (30.9%)	1143 (31.6%)	237 (27.7%)
Sinusitis	967 (19.6%)	814 (20.3%)	153 (16.4%)
Anxiety	871 (17.5%)	712 (17.6%)	159 (16.9%)
Depression	882 (17.7%)	725 (17.9%)	157 (16.8%)
Sleep disturbance	1224 (24.4%)	999 (24.5%)	225 (23.9%)
Allergies (pollen, dust, mold)	1355 (27.0%)	1145 (28.1%)	210 (22.3%)
Chronic back pain	942 (18.9%)	781 (19.3%)	161 (17.1%)
Cancer (any)	871 (17.1%)	771 (18.6%)	100 (10.4%)
Hypertension	854 (17.2%)	617 (15.3%)	237 (25.2%)
High cholesterol	1026 (20.6%)	764 (18.9%)	262 (27.7%)

We report SPRs comparing the prevalence of risk factors, mental health symptoms and health conditions in the FAHS and the NHANES in Table 3. The flight attendants in our study had lower rates of overweight, obesity, and current smoking relative to employed NHANES participants of similar socioeconomic status. Cardiovascular and respiratory outcomes were also decreased among cabin crew relative to the general population. For example, SPRs among female flight attendants were 0.44 (95% CI: 0.39–0.50), 0.53 (95% CI: 0.29–0.96) and 0.82 (95% CI: 0.63–1.07) for hypertension, coronary heart disease, and chronic bronchitis, respectively. Results for cardiac and respiratory outcomes did not change meaningfully when we restricted our analysis to crew with high occupational secondhand smoke exposure (Additional file 1: Table S1).

**Table 3:** Comparative age-adjusted prevalence of health conditions in the FAHS and NHANES

Risk Factors and Health Outcomes	Gender	FAHS count, Unweighted	NHANES count, weighted	Prevalence FAHS, %	Prevalence NHANES, %	SPR (95% CI)
Overweight	Female	1306	768	29.7	59.7	0.50 (0.47–0.53)
	Male	536	1048	44.7	72.6	0.62 (0.59–0.65)
Obesity	Female	304	441	7.1	34.3	0.21 (0.18–0.24)
	Male	113	438	11.1	30.3	0.37 (0.33–0.41)
Current smoker	Female	295	208	7.2	16.1	0.44 (0.38–0.52)
	Male	119	280	12.5	19.4	0.64 (0.56–0.74)
High cholesterol	Female	764	365	14.5	28.4	0.51 (0.46–0.57)
	Male	262	470	18.8	32.6	0.58 (0.52–0.64)
Hypertension	Female	617	332	11.4	25.8	0.44 (0.39–0.50)
	Male	237	384	16.6	26.6	0.63 (0.56–0.70)
Coronary heart disease	Female	42	16	0.67	1.3	0.53 (0.29–0.96)
	Male	24	29	1.5	2.0	0.73 (0.47–1.15)
Chronic bronchitis	Female	239	72	4.6	5.6	0.82 (0.63–1.07)
	Male	42	80	2.9	5.6	0.53 (0.40–0.70)
Asthma	Female	409	226	9.2	17.6	0.52 (0.45–0.61)
	Male	83	191	7.0	13.3	0.53 (0.44–0.63)
Sleep disorder	Female	999	91	21.3	7.1	3.00 (2.45–3.69)
	Male	225	128	17.5	8.9	1.98 (1.64–2.38)
Fatigue	Female	572	87	14.7	6.8	2.18 (1.75–2.70)
	Male	123	61	12.8	4.2	3.04 (2.32–4.00)
Depression	Female	120	17	3.0	13.5	2.23 (1.36–3.66)
	Male	50	13	5.1	0.9	5.57 (3.12–9.94)
Reproductive cancer^a^	Female	267	38	4.9	2.9	1.66 (1.18–2.33)
Any cancer^b^	Female	771	86	14.3	6.7	2.15 (1.73–2.67)
	Male	100	86	5.3	6	0.89 (0.69, 1.14)

*CI* Confidence interval, *FAHS* Flight Attendant Health Study, *NHANES* National Health and Nutritional Examination Survey, *SPR* Standardized prevalence ratio

^a^Breast, uterine, cervical, and ovarian cancers

^b^Breast, ovarian, uterine, cervical, lung, oral, esophageal, prostate, testicular, colon, bladder, melanoma, non-melanoma skin, leukemia, thyroid, brain, lymphoma, liver, kidney, stomach and pancreatic cancers

In contrast, we report a higher prevalence of two broad cancer outcomes in flight crew: “reproductive cancers” (breast, uterine, ovarian, and cervical) and “all cancers” (female reproductive, lung, oral, esophageal, prostate, testicular, colon, bladder, melanoma, non-melanoma skin, leukemia, thyroid, brain, lymphoma, liver, kidney, stomach, and pancreatic). The SPRs for reproductive and any cancers among females were 1.66 (95% CI: 1.18–2.33) and 2.15 (95% CI: 1.73–2.67), respectively, with slightly higher SPRs among those with high occupational secondhand smoke exposure (Additional file 1: Table S1). The SPR for any cancer diagnosis was not elevated among male flight crew overall (SPR = 0.89, 95% CI: 0.69, 1.14), but was among those exposed to occupational secondhand smoke (SPR = 1.70, 95% CI: 1.10–2.63).

We also calculated SPRs for sleep disorders and fatigue and depression symptoms over the past two weeks (Table 4). These conditions were much more prevalent among flight attendants than in the general population. For example, the SPRs for sleep disorders and fatigue among females were 3.00 (95% CI: 2.45–3.69) and 2.18 (95% CI: 1.75–3.69), respectively.

**Table 4:** The relationship between five-year job tenure and the prevalence of diagnosed health outcomes among flight attendants^a^

Condition	Gender	N Cases	Odds Ratio (95% CI)
Sleep disorder	Female	864	0.99 (0.95, 1.04)
	Male	204	1.18 (1.05, 1.31)
Anxiety/depression	Female	902	1.04 (0.99, 1.08)
	Male	193	1.22 (1.08, 1.38)
Alcohol abuse	Female	60	1.25 (1.04, 1.49)
	Male	33	1.37 (1.02, 1.85)
Reproductive cancer	Female	227	1.04 (0.96, 1.11)
Any cancer	Female	678	1.05 (1.00, 1.10)
	Male	84	1.14 (1.00, 1.32)
Hypertension	Female	533	1.01 (0.96, 1.06)
	Male	213	1.05 (0.95, 1.17)
Coronary heart disease	Female	41	1.08 (0.93, 1.26)
	Male	23	0.85 (0.68, 1.06)
Deep vein thrombosis	Female	149	0.98 (0.90, 1.07)
	Male	25	1.03 (0.80, 1.32)
Pulmonary embolism^b^	All	100	1.07 (0.96, 1.20)
	Female	84	1.03 (0.92, 1.16)
Transient ischemic attack^c^	All	32	1.04 (0.86, 1.26)
	Female	26	1.09 (0.88, 1.35)
Peripheral artery disease^d^	All	26	1.33 (1.04, 1.71)
	Female	24	1.37 (1.05, 1.79)
Rheumatoid arthritis	All	100	0.95 (0.85, 1.05)
	Female	85	0.96 (0.86, 1.08)
Osteoarthritis	All	290	1.03 (0.97, 1.09)
	Female	262	1.00 (0.94, 1.07)
Chronic back pain	Female	696	1.04 (1.00, 1.09)
	Male	140	1.02 (0.91, 1.15)
Back surgery	Female	120	1.03 (0.94, 1.14)
	Male	40	1.09 (0.89, 1.33)
Foot surgery	Female	341	1.09 (1.03, 1.16)
	Male	36	1.09 (0.89, 1.33)
Shoulder, elbow, or wrist surgery	Female	309	1.03 (0.96, 1.09)
	Male	57	1.12 (0.95, 1.32)
Chemical sensitivity	Female	331	0.98 (0.92, 1.04)
	Male	30	0.94 (0.74, 1.19)
Multiple chemical sensitivity	All	66	1.08 (0.94, 1.24)
	Female	60	1.08 (0.94, 1.25)
Migraine	Female	570	0.97 (0.92, 1.02)
	Male	52	1.09 (0.89, 1.35)
Hearing loss	Female	444	1.02 (0.97, 1.08)
	Male	75	1.15 (0.98, 1.34)
Eardrum rupture	Female	222	0.99 (0.91, 1.06)
	Male	38	1.10 (0.88, 1.37)
Chronic bronchitis	Female	215	0.96 (0.90, 1.04)
	Male	36	1.12 (0.90, 1.39)
Sinusitis	Female	768	0.98 (0.94, 1.03)
	Male	146	1.17 (1.03, 1.32)

*CI* Confidence interval

^a^All models are adjusted for age, overweight, education, and smoking history

^b^Among participants with high occupational passive smoking exposure, OR = 1.17 (95% CI: 0.90, 1.53)

^c^Among participants with high occupational passive smoking exposure, OR = 1.42 (95% CI: 0.79, 2.55)

^d^Among participants with high occupational passive smoking exposure, OR = 1.54 (95% CI: 0.93, 2.55)

SPRs for all health outcomes were similar when restricting the study populations to non-Hispanic white participants (data not shown). Participants taking the hard copy of the study questionnaire as well as returning 2007 participants did not show meaningful differences in SPRs for the four outcomes examined: bronchitis, coronary heart disease, reproductive cancers, and sleep disorders. However, comparing the entire 2007 wave of the FAHS to the 2013–2014 NHANES participants yielded elevated SPRs for bronchitis and heart disease (data not shown).

We found associations between each five-year increase in net job tenure and sleep disorders (OR = 1.18, 95% CI: 1.05, 1.31) and anxiety/depression (OR = 1.22, 95% CI: 1.08, 1.38) among male participants, and with alcohol abuse for both genders (female: OR = 1.25, 95% CI: 1.04, 1.49; male: OR = 1.37, 95% CI: 1.02, 1.85) (Table 4). Job tenure was also related to the prevalence of any cancer among males (OR = 1.14, 95% CI: 1.00, 1.32) and females (OR = 1.05, 95% CI: 1.00, 1.10), and to peripheral artery disease among females (OR = 1.37, 95% CI: 1.05, 1.79). Net tenure was related to sinusitis among males (OR = 1.17, 95% CI: 1.03, 1.32), and to foot surgery (OR = 1.09, 95% CI: 1.03, 1.16) among females (Table 4). Chronic back pain was marginally related to tenure among females (OR = 1.04, 95% CI: 1.00, 1.09). Because we are particularly interested in the effects of secondhand smoke on the prevalence of pulmonary embolism, peripheral artery disease, and transient ischemic attacks, we conducted a *post-hoc* analysis evaluating associations between tenure and these health outcomes among flight crew working prior to 1988; the ORs for pulmonary embolism and peripheral artery disease rose to 1.17 (95% CI: 0.90, 1.53) for males and 1.53 (95% CI: 0.93, 2.55) for females, and the OR for transient ischemic attack among females was 1.42 (95% CI: 0.79, 2.55).

Finally, we evaluated associations between five-year job tenure prior to age 45 years and several reproductive and perinatal outcomes: miscarriage, preterm birth, fetal abnormality, and infertility (Table 5). These conditions were all related to job tenure, with ORs of 1.33 (95% CI: 1.20, 1.46), 1.43 (95% CI: 1.17, 1.74), 1.64 (95% CI: 1.23, 2.18), and 1.45 (95% CI: 1.29, 1.64), respectively. The association between job tenure and infertility was attenuated slightly when restricting to parous women, but remained elevated at an OR of 1.34 (95% CI: 1.18, 1.52).

**Table 5:** The relationship between five-year job tenure prior to age 45 and prevalence of reproductive and perinatal outcomes among female flight attendants^a^

Condition	N Cases	Odds Ratio (95% CI)
Preterm birth	74	1.43 (1.17, 1.74)
Miscarriage	367	1.33 (1.20, 1.46)
Fetal abnormality	29	1.64 (1.23, 2.18)
Infertility^b^	272	1.45 (1.29, 1.64)

^a^All models are adjusted for age, overweight, education, and smoking history

^b^Among parous women: OR = 1.34, 95% CI:, 1.18, 1.52

## Discussion

To our knowledge, we have conducted the largest study characterizing the overall health of flight attendants relative to the general population. Consistent with previous studies, we report a higher prevalence of fatigue, depression, anxiety, and sleep disorders, as well as reproductive and all cancers. This is striking given lower observed rates of overweight, smoking, and chronic respiratory and cardiovascular conditions among flight crew. We also report associations between job tenure and reproductive, perinatal, mental health, and cancer outcomes, as well as peripheral artery disease and sinusitis. Our study informs future research priorities regarding the health of this understudied group of workers, and raises the question of what can be done to minimize the adverse exposures and health outcomes common among cabin crew.

Our finding of a greater prevalence of reproductive and all cancers among flight crew is consistent with most of the epidemiologic literature on this topic [20]. We also observed that job tenure was associated with prevalence of cancer at all sites among males and females. While a recent study reported no evidence of increased breast cancer or melanoma mortality within a large cohort of U.S. flight attendants relative to the general population, this study was limited by reliance on cancer mortality rather than incidence data for cancers that have relatively low mortality rates, and by a short median employment tenure of 5.9 years [21]. The latter is problematic given the long induction and latency periods of cancers, especially solid tumors [22]. In contrast, the median tenure among participants in the FAHS was 19 years. Our results are also consistent with flight crews’ occupational exposures to ionizing radiation [2, 8], circadian rhythm disruption and resulting sleep disorders [3], historical exposures to secondhand smoke [5], and ongoing exposures to other chemical agents [4, 5], most of which are classified as confirmed or probable carcinogens in humans [23–25]. While beyond the scope of the study reported here, we plan to evaluate a wide range of specific cancers in a future investigation.

We report increased prevalence of adverse sleep and mental health outcomes among flight crew. We also observed associations between tenure, anxiety/depression, sleep disorders, and alcohol abuse. Our results are consistent with the existing literature, though ours is the only study population to have evaluated all of these conditions [3, 13]. Studies also report elevated rates of suicide among cabin crew [21]. Previous research suggests risk factors for adverse mental health outcomes among flight crew, including long or irregular working hours, sexual harassment, and a lack of employer protections with respect to occupational exposures [26]. Flight attendants also anecdotally report work-related disruptions to their dietary intake and nutritional patterns, in terms of both meal timing and availability of nutritious food while traveling, which could potentially affect multiple health outcomes as well. Furthermore, it should be noted that sleep disorders, which can be related to Circadian rhythm disruption, are independent risk factors for adverse mental health outcomes, including suicide [27, 28].

We observed positive associations between tenure as a flight attendant acquired during a woman’s reproductive years and infertility, miscarriage, preterm birth, and fetal abnormalities. Our results are consistent with the handful of studies that have examined reproductive and pregnancy outcomes among flight crew and passengers [11, 29]. For example, a recent high-quality study observed associations between rates of miscarriage and circadian rhythm disruption, cosmic ionizing radiation exposure, and high physical job demands among flight attendants [11]. Our findings are also consistent with research linking adverse pregnancy outcomes to ionizing radiation exposure, shift work and physical job demands within other occupations [30–32]. Cabin crew have the largest annual ionizing radiation dose of all U.S. workers (e.g. 3.07 mSv vs. 0.59 mSv for U.S. Department of Energy workers) [8], and can easily exceed the prenatal ionizing radiation exposure guidelines released by the NCRP or the International Commission on Radiological Protection [8, 33]. These exposures, which are not regulated among flight crew, may be particularly problematic for pregnant workers.

We report a lower prevalence of respiratory and cardiac conditions among flight crew relative to the general population, overall and when restricting to workers with high occupational secondhand smoke exposures prior to 1988. This contrasts with our findings from the previous wave of the FAHS [14]. While flight attendants in the 2007 wave reported lower rates of hypertension and asthma compared to the general population, they reported considerably higher rates of chronic bronchitis and heart disease. Flight attendants’ occupational exposures, such as noise and circadian rhythm disruption, have also been associated with cardiovascular outcomes in other study populations [34, 35]. Furthermore, previous studies have reported associations between employment as a flight attendant and chronic bronchitis, though these were limited by low sample size, response rate, or a lack of participant blinding to study hypotheses [9]. We considered several explanations for our unexpected results, such as differences between hard copy and online questionnaire takers, 2007 and 2014–2015 FAHS participants, and the 2005–2008 and 2013–2014 NHANES study populations. Sensitivity analyses indicated that (1) chronic bronchitis and coronary heart disease rates were higher in the 2007 wave of the FAHS relative to the 2013–2014 NHANES participants, ruling out changes in prevalence within the NHANES as a contributing factor, (2) results were robust when restricting analyses to participants who filled out a hard copy questionnaire, and (3) 2007 participants who returned for the 2014–2015 wave had reduced rates of chronic bronchitis and heart disease. Given these findings, we suggest two possibilities explaining the difference in results between the waves of the FAHS, though we cannot rule out the possibility of random chance. First, working populations often exhibit lower mortality and morbidity rates than the general population due to the healthy worker effect [36]. This occurs because people with significant health issues may be unable to maintain employment, especially when the job is physically demanding. The resulting selection bias can obscure associations between occupational exposures and health outcomes. Chronic bronchitis and coronary heart disease are both severe and often deteriorating conditions that would significantly impair a flight attendant’s ability to perform their job duties, and participants reporting these diagnoses in 2007 may not have continued to work until 2015. Secondly, more time has elapsed since smoking bans were instituted on flights, and the risk of adverse respiratory and cardiac outcomes generally declines continually in the years after cessation of cigarette smoke exposure [37]. Hence, one can expect to see fewer incident cases of smoking-related bronchitis and heart disease among flight crew as time goes on, with a simultaneous increased rate of attrition for participants with these health outcomes due to an inability to sustain job demands. Interestingly, tenure as a flight attendant was related to the prevalence of peripheral artery disease in our study, especially among workers exposed to high levels of secondhand tobacco smoke. This is consistent with reports that former smoking has a more persistent effect on peripheral artery disease risk than on the risk of coronary heart disease [38].

Participants in the FAHS report a relatively high prevalence of sinonasal, ear and musculoskeletal symptoms, consistent with previous studies [2, 9, 14]. We also found positive associations between job tenure and sinusitis among males, foot surgery among females, and a marginal association with chronic back pain among females. Reasons underlying gender-specific associations are unclear, and these results should be considered preliminary given their lack of precision. However, the observed associations are consistent with the presence of respiratory irritants in the cabin environment, and with flight attendants’ physical job demands and exposures to noise and changes in barometric pressure [2, 4]. Past studies have also identified work-related psychosocial factors related to musculoskeletal disorders among flight crew [39], which include psychological job demands, harassment, and job insecurity.

Limitations of our study include its cross-sectional design, which precludes inferences about causality. In addition, health outcomes in our study and in the NHANES were based on self-report; validation through medical records was not possible due to the scope and cost of this endeavor. Validity of self-reported health outcomes varies by study population and the outcome of interest. Sensitivity and specificity of self-reported outcomes relative to medical records or linkage to disease registries were found to be moderate to high for cancer, musculoskeletal disorders and mental health diagnoses, including in the NHANES [40–42]. Validity is often further improved among those with higher socioeconomic status [40]. Because flight attendants may differ from a representative sample of the general population in ways that could affect health, we restricted NHANES respondents to adults of comparable socioeconomic status (as measured by family income to poverty ratio, educational attainment, and employment status) in order to make the two cohorts more comparable. Nevertheless, we recognize that further health-related differences may exist between flight attendants and even a restricted general population survey. Hence, future studies should compare flight attendant health with that of U.S. workers in similar occupations, such as nursing or service industry professions.

An additional limitation of our study is the use of an online recruitment strategy, meaning that our response rate and the representativeness of our study with regard to the total population of U.S. cabin crew are unclear. Other limitations include reliance on job tenure as a surrogate for occupational exposures, lack of correction for multiple testing, and the use of a uniform set of potential confounders for evaluating all health outcomes. The goals of our study were to characterize the health of flight attendants relative to the general population and to identify future research directions. We plan to evaluate specific exposures and identify potential confounders for individual exposure-outcome analyses in future research efforts.

Strengths of our study include access to the resources of a large cohort of flight crew with information on a range of health outcomes, work experiences, and potential confounders. Our study presents the most comprehensive profile of cabin crew health to date, and the multiple waves of our cohort allow us to describe changes in the health of U.S. cabin crew over time. In addition, online questionnaires are an increasingly popular option in epidemiologic research, including high profile studies such as the Millennium Cohort and the Nurses’ Health Study 3 [43]. This mode of data collection allows for validation checks, reduced data entry and coding errors, personalized question administration, convenience to participants, equal or better validity compared to hard copy questionnaires, and the collection of metadata, such as date, time, and time to completion, which can be used for quality control and sensitivity analyses [43].

Our study findings contribute to the sparse literature on flight attendant health, which may also be applicable to passengers, especially frequent flyers or vulnerable subpopulations such as the elderly, those with preexisting health conditions, and pregnant women. Conducting high quality studies within this group of workers is important given the fact that U.S. flight crew are subject to fewer protections than most workers in this country and relative to flight attendants working in the European Union (EU). For example, the EU requires airlines to monitor radiation dose, organize schedules to reduce radiation exposure (e.g by rationing flight routes with higher radiation exposures, such as international or circumpolar flights), and inform workers of current studies and health risks [44]. Furthermore, studies consistently report fatigue and reduced mental health among cabin crew, which can affect worker quality of life and passenger safety. Possible interventions for improving quality of life among flight crew include altering the organization of work hours, providing more opportunities for rest and education regarding best sleep practices, and workplace policies for reducing sexual harassment [39].

Future studies should address the healthy worker effect through statistical methods. A more comprehensive assessment of cancer rates among cabin crew is warranted as well. It is also important to note that the lower prevalence of cardiac and respiratory disease among our participants does not rule out the possibility, even in the FAHS, of in-flight exposures being related to more subtle health markers, such as fluctuations in heart rate variability or reductions in pulmonary function, which could be important among susceptible crew and passengers. Hence, future studies could monitor subclinical cardiopulmonary changes in relation to flight.

## Conclusions

To our knowledge, we have conducted the largest study of general flight crew health to date. Despite a strong observed healthy worker effect, we report that flight attendants have elevated rates of reproductive cancers, cancer at all sites, sleep disorders, and mental health conditions relative to the general U.S. population. The prevalence of adverse reproductive and perinatal outcomes, mental health and sleep disorders, musculoskeletal conditions, all cancers, peripheral artery disease, and sinusitis were related to tenure as a flight attendant. Our results provide new information to guide future research regarding the health of this understudied group of workers.

## Additional file

Additional file 1: **Table S1**. Comparative age-adjusted prevalence of health behaviors and conditions in the Harvard Flight Attendant Health Study (FAHS, 2014–2015) and NHANES (2013–2014), evaluating only flight attendants with occupational smoking exposure prior to the year 1988. (DOCX 21 kb)

## Abbreviations

CI: Confidence interval
EU: European Union
FAHS: Flight Attendant Health Study
NCRP: National Council on Radiation Protection
NHANES: National Health and Nutrition Examination Survey
OR: Odds ratio
SPR: Standardized prevalence ratio
