# Supplementary material for: Estimating the health consequences of flight attendant work: comparing flight attendant health to the general population in a cross-sectional study
Source: BMC Public Health. 2018 Mar 23;18:346. doi: 10.1186/s12889-018-5221-3 (PMC5865289; doi:10.1186/s12889-018-5221-3)
Supplement: Supplementary file 1 — Table S1. Comparative age-adjusted prevalence of health behaviors and conditions in the Harvard Flight Attendant Health Study (FAHS, 2014–2015) and NHANES (2013–2014), evaluating only flight attendants with occupational smoking exposure prior to the year 1988. (DOCX 21 kb) [file 12889_2018_5221_MOESM1_ESM.docx]

Additional file 1 Table S1 Comparative age-adjusted prevalence of health behaviors and conditions in the Harvard Flight Attendant Health Study (FAHS, 2014-2015) and NHANES (2013-2014), evaluating only flight attendants with occupational smoking exposure prior to the year 1988.

|  |  |  | |  |  |  |  |  |
| --- | --- | --- | --- | --- | --- | --- | --- | --- |
| Risk Factors and  Health Outcomes | Gender | FAHS count,  unweighted | NHANES count, weighted | | | Prevalence  FAHS, % | Prevalence  NHANES, % | SPR (95% CI) |
| Overweight | Female | 460 | 768 | | | 18.6 | 59.7 | 0.31 (0.28-0.35) |
|  | Male | 125 | 1048 | | | 31.6 | 72.6 | 0.43 (0.36-0.53) |
| Obesity | Female | 116 | 441 | | | 4.8 | 34.3 | 0.14 (0.11-0.18) |
|  | Male | 23 | 438 | | | 4.4 | 30.3 | 0.14 (0.08-0.26) |
| Current smoker | Female | 77 | 208 | | | 3.1 | 16.1 | 0.19 (0.14-0.26) |
|  | Male | 16 | 280 | | | 4.6 | 19.4 | 0.24 (0.13-0.43) |
| High cholesterol | Female | 398 | 365 | | | 13.9 | 28.4 | 0.49 (0.42-0.57) |
|  | Male | 68 | 470 | | | 17.5 | 32.6 | 0.54 (0.40-0.71) |
| Hypertension | Female | 327 | 332 | | | 10.7 | 25.8 | 0.42 (0.35-0.49) |
|  | Male | 73 | 384 | | | 17.7 | 26.6 | 0.67 (0.50-0.89) |
| Coronary heart disease | Female | 29 | 16 | | | 0.76 | 1.3 | 0.60 (0.29-1.25) |
|  | Male | 8 | 29 | | | 2.1 | 2.0 | 1.01 (0.39-2.62) |
| Chronic bronchitis | Female | 99 | 72 | | | 3.7 | 5.6 | 0.66 (0.47-0.93) |
|  | Male | 17 | 80 | | | 4.0 | 5.6 | 0.72 (0.37-1.39) |
| Asthma | Female | 143 | 226 | | | 6.0 | 17.6 | 0.34 (0.27-0.43) |
|  | Male | 19 | 191 | | | 4.8 | 13.3 | 0.36 (0.20-0.64) |
| Sleep disorder | Female | 395 | 91 | | | 16.7 | 7.1 | 2.36 (1.88-2.96) |
|  | Male | 63 | 128 | | | 16.6 | 8.9 | 1.87 (1.34-2.61) |
| Reproductive cancer^1^ | Female | 151 | 38 | | | 5.2 | 2.9 | 1.76 (1.20-2.56) |
| Any cancer^2,3^ | Female | 424 | 86 | | | 15.3 | 6.7 | 2.29 (1.81-2.89) |
|  | Male | 44 | 86 | | | 10.1 | 6 | 1.70 (1.10, 2.63) |

CI: confidence interval; SPR: standardized prevalence ratio

1. Breast, uterine, cervical, and ovarian cancers.

2. Breast, ovarian, uterine, cervical, lung, oral, esophageal, prostate, testicular, colon, bladder, melanoma, non-melanoma skin, leukemia, thyroid, brain, lymphoma, liver, kidney, stomach and pancreatic cancers.
